# Contemporary review of primary membranous nephropathy

**DOI:** 10.3389/fimmu.2026.1836347

**Published:** 2026-06-19

**Authors:** Edward J. Filippone, John L. Farber

**Affiliations:** 1 Division of Nephrology, Department of Medicine, Sidney Kimmel Medical College at Thomas Jefferson University, Philadelphia, PA, United States; 2 Department of Pathology, Sidney Kimmel Medical College at Thomas Jefferson University, Philadelphia, PA, United States

**Keywords:** membranous nephropathy, NELL-1, obinutuzumab, phospholipase A2 receptor antibodies, rituximab, thrombospondin type-1 containing domain 7A

## Abstract

Primary membranous nephropathy (PMN) is a single-organ autoimmune disease caused by autoantibodies targeting podocyte-associated antigens, most commonly phospholipase A2-receptor (PLA2R). Although 14 other antigens have been identified, the target remains unidentified in 5 – 10%. Specific secondary causes have been associated with each antigen with much overlap. PMN usually presents as nephrotic syndrome. About one-third spontaneously remit, one-third progress to ESKD, and the rest maintain non-remitting proteinuria. Treatment includes supportive anti-proteinuric therapy. Immunosuppression is guided by KDIGO-based risk stratification. Low-risk cases can be watched expectantly. Very high-risk cases (declining eGFR, complications of nephrosis) should receive alternating steroids/cyclophosphamide. Moderate to high-risk cases should receive rituximab, expecting 60 – 80% response. PLA2R-titers can be followed to assess response in positive cases. Immunologic remission precedes clinical remission by months. Reemergence of antibodies signifies impending relapse. Causes of rituximab resistance include reduced bioavailability, anti-rituximab antibodies, and chronic scarring despite immunologic remission. The latter precludes further immunosuppression. Reduced bioavailability may respond to redosing or use of the more potent B-cell depleters, obinutuzumab or ofatumumab, neither of which cross react with anti-rituximab antibodies. The roles of chimeric-autoantibody-effector-cell therapy, APRIL/BAFF inhibition, anti-plasma-cell therapy, or complement inhibition remain to be determined. Transplantation is optimal therapy for ESKD. Recurrence is common. In PLA2R-positive cases, antibody reemergence or titers failing to decrease post-transplantation indicates protocol biopsy. Recurrence is not likely to remit and should prompt consideration of rituximab.

## Introduction

Membranous nephropathy (MN) is the most common cause of the nephrotic syndrome, excluding diabetes in Caucasian adults (1), with an incidence of about 6–10 per million population. About 80% are kidney limited, termed primary membranous nephropathy (PMN) and considered a single organ autoimmune disease. Secondary causes include infections, systemic illnesses, and drug/toxin exposures (see **Table 1**) (1). Antibodies target an antigen expressed in podocytes to form immune complexes on the outer surface of the glomerular basement membrane (GBM). Complement is then activated predominantly by the classical pathway (2), although both the alternate pathway (3) and lectin pathways (4) may contribute to variable degrees (5). The membrane attack complex is formed, podocytes are injured, and proteinuria results from a disrupted filtration barrier.

**Table 1:** Secondary causes of membranous nephropathy (from most to least frequently associated).

Category	Examples
Infections	Hepatitis B and C HIV Chronic bacterial infections Malaria Schistosomiasis Syphilis Post-streptococcal glomerulonephritis
Autoimmune diseases	Systemic lupus erythematosus Rheumatoid arthritis* Mixed connective tissue disease Sarcoidosis Sjogren’s syndrome CIDP Others rarely (systemic sclerosis, thyroiditis, urticarial vasculitis, ankylosing spondylitis, MPO-ANCA vasculitis, IgG4-RD)
Medications	Penicillamine Gold bucillamine (91) Mercury containing compounds TIMs (92) skin lightening creams (93) NSAIDs Other medications containing a sulfhydryl group lipoic acid (89), tiopronin (90) (91) (92) (93)
Malignancy	Solid tumors Lung, prostate, colorectal, breast, gastric, and esophageal Hematologic malignancies Non-Hodgkin’s lymphoma CLL (usually membranoproliferative GN) Hodgkin’s disease (usually minimal change disease) Paraneoplastic (unknown primary)

CIDP, chronic inflammatory polyneuropathy; CLL, chronic lymphatic leukemia; HIV, human immunodeficiency virus; IgG4-RD, IgG4-related disease (216); MPO, myeloperoxidase; NSAID, non-steroidal anti-inflammatory drug; TIMs, traditional indigenous medications.

*mainly via AA amyloidosis or medications, mesangial proliferative glomerulonephritis less common.

PMN occurs at all ages, but most cases are middle-aged with peak incidence at ages 30–50 years-old (6). Patients usually present the with nephrotic syndrome, normotension, and preserved GFR. About 20% have reduced GFR at presentation (6). About 20% have only sub-nephrotic range proteinuria with excellent prognosis; however, close follow-up is required, as up to 60% of these may develop nephrotic range proteinuria, often within the first year and with a faster decline of kidney function (7). Microscopic hematuria is common. Thromboembolism may occur, especially with severe nephrosis, and may involve the renal veins. PMN typically evolves over years to a decade or more. Approximately one-third of nephrotic patients will have spontaneous remission and about one-third will progress to ESKD, with the remainder maintaining some degree of proteinuria (1).

The pathology of PMN is characteristic (6). By light microscopy (LM), the picture is variable. Initially, glomeruli may appear normal. With progression, the GBM widens from subepithelial deposits with GBM spikes in between the deposits best visualized with the Jones methenamine-silver stain. Eventually, the GBM will encompass the deposits which may appear as pinholes on silver stain. Immunofluorescence microscopy (IFM) demonstrates granular deposits of IgG (predominantly subclass IgG4) and C3 diffusely covering glomerular capillary walls without mesangial staining. C1q is usually absent. On electron microscopy (EM), electron dense deposits are found in the subepithelial space with no appreciable mesangial or subendothelial deposits.

## Purpose and methodology

The purpose of this narrative review is to provide updated information on the pathophysiology, clinical classification, and approach to treatment of PMN considering recent advances. The antigenic targets of the pathogenic autoantibodies will be outlined. Treatment with B-cell depletion will be discussed in detail. PubMed was interrogated up to February 1, 2026 for relevant articles. The reference lists of articles available were also reviewed for possible additional studies. Studies considered most appropriate by the authors have been selected for inclusion.

### Phospholipase A2 receptor antibodies and primary membranous nephropathy

The classification of MN into primary versus secondary is moving towards classification based on identification of the target podocyte antigen. In 2009 the phospholipase-A2 receptor 1 (PLA2R) was identified as the target antigen in 70 – 80% of PMN cases, and many subsequent studies assessed various aspects of its measurement.

Circulating PLA2R antibodies can most sensitively be detected by Western blot (WB) and can be quantitated by ELISA. A semiquantitative indirect immunofluorescence test (IIF) utilizing PLA2R-expressing HEK293 cells is more sensitive and specific than ELISA and can be used to verify a marginally positive ELISA. Both ELISA and IIF are commercially available (EUROIMMUN assay, Revvity, Inc., Waltham, MA). The EUROIMMUN ELISA is considered positive by the manufacturer when ≥ 20 Relative Units (RU)/ml; < 14 RU/ml is considered negative and ≥ 14–20 is considered borderline. Other cutoffs have for positivity (e.g., 2 RU/ml and 14 RU/ml) been used in various studies with true negativity often considered at < 2 RU/ml (*vide infra*).

The current gold standard for diagnosing PLA2R-positive PMN is antigen detection in the glomerular capillary wall by immunofluorescence, immunohistochemistry, or laser microdissection/mass spectrometry (LM/MS) (8). All biopsies with PMN should be evaluated for tissue PLA2R-positivity. Supportive pathological features include IgG4 dominance, limited staining for other reactants (C1q, IgA, IgM), and global/diffuse deposits restricted to glomerular capillary loops (not mesangial) (9).

Although PLA2R positivity is often considered synonymous with PMN, tissue positivity for PLA2R is not specific (9). Nearly two-thirds of MN associated with either hepatitis B (10) or C (11) may be PLA2R-tissue positive with renal response to antiviral therapy. One-half of HIV-associated MN may be PLA2R-tissue positive, usually with undetectable viral loads on anti-retroviral therapy (12). Nearly one-half of syphilis-associated MN may be PLA2R-tissue positive (13). Malignancy may be found in 5% of PLA2R-tissue positive cases (11). Half or more cases of sarcoidosis-associated MN may be PLA2R-tissue positive with either active (14, 15) or quiescent (14) sarcoid disease.

Discrepancies between serologic assays and tissue staining occur. Both false-negative and false-positive serum ELISA reactions occur, and tissue staining is considered the gold standard. A positive PLA2R tissue stain with negative serology suggests that either it is early in the course of disease with all antibodies binding to kidney tissue, or it is later with development of immunologic remission. Supporting the former are reports of PLA2R-positive glomerular staining with negative serological testing (both ELISA and IIF) that turned serologically positive on serial testing (16, 17). In support of the latter, data show a more benign course with PLA2R-negative serology, suggesting that some serologically negative patients spontaneously entered immunologic remission (18, 19).

Positive serology may occur with a negative tissue stain. Either the serologic assay is a false-positive, or the tissue stain is false-negative. Luo et al. found that 19 of 229 serologically positive patients (8.3%) were negative for tissue antigen by immunofluorescence. However, all 19 tested positive by immunohistochemistry (20). Notably, only 5 of 19 were diffusely, globally, and strongly positive, whereas 14 were either focally and segmentally or weakly positive. The immunofluorescence-negative immunohistochemistry-positive patients had higher serum antibody levels and more severe clinical manifestations than the immunofluorescence-positive group suggesting that reactive epitopes on PLA2R may be masked by bound antibody and are exposed by heating during preparation for immunohistochemistry. Zand et al. identified 7 out of 250 (2.8%) MN patients with negative tissue immunofluorescence for PLA2R that had PLA2R detected by LM/MS, 2 of which were serologically positive (21). Hence, if tissue immunofluorescence-negative MN is found in a serologically-positive patient, either immunohistochemistry or LM/MS should be performed to rule out false-negative tissue immunofluorescence.

False-positive serum ELISA reactions occur proven by an alternative diagnosis found on biopsy. Hoxha et al. reported a nephrotic patient with ELISA levels between 120 and 258 RU/ml that had 2 biopsies showing diabetic nephropathy (22). The antibodies reacted against the polyhistidine tag and not PLA2R itself. Both IIF and western blot serum assays were negative. Similarly, Caza and Larsen reported 2 patients with ELISA levels ranging from 85.7 to 182.9 RU/ml that had diabetic nephropathy on biopsy; an IIF assay was negative in both (23). Two patients from the NEPTUNE database had ELISA values of 132.5 RU/ml and 22.5 RU/ml with negative serum IIF and Western blots that had FSGS and MCD, respectively (24). Hence false-positive ELISAs are typically accompanied by negative IIF and WB. Detectable antibodies can also precede clinical disease by months precluding them as false-positive (25).

A positive serology may obviate the need for a biopsy. A very high level of specificity (>99%) would be necessary. Lowering the cutoff for positivity from the manufacturer recommended level of ≥ 20 RU/ml to ≥ 2 RU/ml improves sensitivity with loss of specificty (26–28). An elevated ELISA does not prove PMN, secondary causes must still be excluded. Evaluating 132 anti-PLA2R-positive patients (defined as > 2 RU/ml with a positive IIF assay), Bobart et al. found 35 had secondary causes (11 autoimmunity, 15 cancer, 3 medications, and 6 paraproteins) (28). The remaining 97 were divided based on eGFR of 60 (all eGFR in ml/min/1.73m^2^). All 60 patients with eGFR > 60 had MN with no features of secondary MN, and only 2 had superimposed lesions (diabetic nephropathy, FSGS). All 37 with eGFR < 60 also had MN, but 7 had additional lesions, 2 being actionable (interstitial nephritis, crescentic GN), suggesting biopsy is indicated with a lower eGFR.

Bobart et al. subsequently described 163 additional patients that had positive serology and a biopsy, of which 47 had secondary causes (17 autoimmunity, 10 malignancy, 6 paraproteinemia, 7 medication-related) and 15 had diabetes mellitus (29). The remaining 101 had MN on biopsy with only one having an associated finding potentially altering therapy (interstitial nephritis).

By contrast, Zee et al. analyzed 468 proteinuric patients (> 0.5 g/day or g/g creatinine) that had a kidney biopsy plus serologic analysis for anti-PLA2R antibodies; 85 had MN (24). Importantly, this study included patients with non-nephrotic proteinuria. All secondary causes of MN were not systematically excluded, however, except systemic lupus. Combining an ELISA ≥ 2 RU/ml with a positive IIF gave a sensitivity of 60%, specificity of 100%, and a positive predictive value of 100% for MN. However, analyzing just nephrotic patients, 13 of 27 (48%) with eGFR >60 and 9 of 11 (82%) with eGFR <60 had at least one additional biopsy feature, some of which potentially could have altered therapy. Notably, 2 had ELISA ≥ 20 RU/ml with negative IIF, and both had other lesions on biopsy (FSGS, MCD).

Ragy et al. performed a systematic review and meta-analysis of studies assessing the ability of biomarkers to obviate biopsy (30). Thirty-eight studies evaluated the EUROIMMUN ELISA and 27 the EUROIMMUN IIF assays. The pooled sensitivity/specificity of the ELISA was 0.64/94.7% at a cutoff of 20 RU/ml and of the IIF was 0.69/98% at 1:10 dilution. Regarding false-positives, 117 of 1, 015 positive patients (~11%) from 17 studies were false-positives for PMN, due mainly to secondary causes (hepatitis B, malignancy, and systemic lupus) with occasional other glomerulopathies found. They conclude that after ruling out secondary causes, a EUROMMIN ELISA ≥ 20 RU/ml can obviate a biopsy.

In our opinion, biopsy can be avoided in a PLA2R-positive patient if both ELISA and IIF are positive, nephrotic syndrome is present, eGFR is preserved (> 60), the patient is not diabetic, and secondary causes of MN have been thoroughly excluded.

### Prognosis in PLA2R-positive membranous nephropathy

Patients with non-nephrotic proteinuria have an excellent prognosis but do require close follow-up. For nephrotic patients, attaining remission markedly improves prognosis (31, 32). Complete remission carries an excellent prognosis, is a valid surrogate endpoint, and is associated with a low risk of relapse to nephrotic syndrome or progression to ESKD. However, the frequency with which relapses to sub-nephrotic levels of proteinuria occur is ill-defined. Partial remission is also associated with improved outcomes but is associated with relapses into nephrotic syndrome and is considered only a reasonably likely surrogate.

The ability to predict remission, partial or complete, either spontaneously or in response to immunosuppression, would facilitate personalized therapy. Numerous factors have been evaluated, either at baseline or over time, to determine the need for immunosuppression, and if initiated, when therapy is ineffective and should be stopped or switched to an alternative agent.

In most studies, higher baseline ELISA PLA2R-antibody titers (e.g., > 150 RU/ml, although KDIGO uses a cutoff of > 50 RU/ml to define high risk) correlated with disease severity, including higher levels of proteinuria (18), lower serum albumin (18, 33), and lower eGFR (34). Also, higher baseline titers are associated with lower chance of spontaneous remission and higher chance for not responding to immunosuppression (18, 35). Many studies indicate immunologic remission (disappearance of PLA2R antibodies) precedes clinical remission by months (6, 18, 36) and the antibody trend can be followed as a predictor of subsequent clinical remission (spontaneous or treatment induced) (37). Reemergence of antibodies after immunologic remission predicts subsequent relapse (18).

Beck et al. first looked at the relationship between decline in anti-PLA2R antibodies in response to therapy (rituximab) and the clinical outcome (38). They found that 17 of 25 (68%) PLA2R-positive patients had either a decline or disappearance by 12 months, a response that correlated significantly with clinical remission. The disappearance of antibodies preceded remission by months, and the greatest correlation between decrease in antibody titer and subsequent clinical outcome occurred at 3 months.

Others confirmed these relationships. Hoxha et al. prospectively followed 133 patients and found that antibody reduction occurred earlier than proteinuria reduction, especially in those immunosuppressed (36). Baseline PLA2R antibody levels were lower in those attaining remission by 12 months, and higher baseline levels signified a longer time to remission. Levels fell over time among patients achieving spontaneous remission but remained elevated in those without remission. Hofstra et al. found a significant correlation between antibody titer and baseline proteinuria in 82 nephrotic patients; spontaneous remission occurred more frequently in the lowest tertile than the highest tertile (38% versus 4%) (39).

Ruggenenti et al. studied 81 patients given rituximab and found that lower baseline titers and full antibody depletion at 6 months strongly predicted remission (18). Similarly, Wang et al. studied 94 patients given tacrolimus and found higher baseline levels were significantly associated with reduced chance for remission (33).

Guo et al. developed a nomogram for predicting immunologic remission at 6 months in PLA2R-antibody-positive Chinese patients in response to rituximab that included PLA2R-antibody titers, hemoglobin level, and gender (40). Geng et al. developed a nomogram for predicting failure to attain remission at 12 months in PLA2R-antibody positive Chinese patients based on PLA2R-antibody titer, interstitial inflammation, and C3 deposition (41). Wei et al., also studying Chinese patients, found pathologic features superior to clinical features in predicting response to immunosuppression (42). These nomograms have not been validated in other populations or used in randomized controlled trials (RCTs).

Barbour assessed the decline of anti-PLA2R antibody levels in conjunction with clinical variables at 3 landmark times (baseline, 3-, and 6-months) for predicting remission at 12-months (43). By multivariable analysis, baseline anti-PLA2R titers > 320 RU/ml and 6-month titers > 14 RU/ml were each significantly associated with no remission. However, the best predictive model combined the 3-month change from baseline in both PLA2R antibody levels (decline) and serum albumin (increase) compared to just considering antibody decline alone.

Expanding on this, Barbour et al. studied 187 PLA2R-positive patients (cutoff ≥ 14 RU/ml) to develop predictive equations for 12-month remission based on variables at baseline, 3-, and 6-months (44). Both the 3- and 6-month models performed better than baseline, with little difference between them. Both the 3- and 6-month models included change from baseline in PLA2R-antibody levels. Changes in both proteinuria and PLA2R antibody levels were independently associated with remission, such that very large changes in either one could offset minimal changes in the other. The results were consistent regardless of immunosuppression use. A low probability of remission at 3-months suggests the need to immunosuppress if on conservative therapy or the need to alter immunosuppression (additional dosing or switch to alternative agent). Conversely, a higher probability of remission suggests staying the current course. Of note, remission can clearly occur after 12 months, which would not be predicted by these equations.

In antibody-positive patients being immunosuppressed, levels should be measured at 3 and 6 months (45). Disappearance of antibodies by 6 months strongly predicts remission and can prompt discontinuation of immunosuppression (46). Continued monitoring every 3 to 6 months for re-emergence is advisable in such cases (6). Failure to clear antibodies at 6 months suggests considering further immunosuppression (if declining) or switching to an alternative agent if not declining (46).

In addition to the amount of proteinuria and regardless of PLA2R status, urinary excretion of low-molecular-weight proteins, such as β2-microglobulin and α1-microglobulin, has been evaluated as a prognostic marker in PMN, as their enhanced excretion reflects tubular damage (47–50). Also, selectivity of proteinuria, i.e., the ratio of clearances of a larger protein (IgG) to an intermediate-sized protein (transferrin), may reflect glomerular damage and have prognostic importance in MN (47, 48). These assays currently are not widely available, and KDIGO recommends their measurement (46) for prognosis as valuable but optional in addition to the degree of proteinuria, eGFR, and anti-PLA2R levels.

### PLA2R antibodies and epitope spreading

PLA2R is a transmembrane glycoprotein with an extracellular portion consisting of an N-terminal cysteine-rich domain (CysR), a fibronectin type-II domain (FNII), and 8 consecutive C-type lectin domains (CTLD1-8). Several groups investigated the specific epitope(s) reactive to PLA2R antibodies. Kao et al. using a panel of PLA2R constructs localized the major epitope to the first 3 N-terminal domains (CysR, FNII, and CTLD1) and concluded these domains represent the smallest portion of the molecule retaining antigenicity (51). Fresquet et al. similarly found reaction to the CysR, FNII, and CTLD1–3 fragment. However, using different methodology, they determined the CysR domain alone contained the dominant epitope.

Seitz-Polski found reactivity to PLA2R segments beyond CysR, specifically CTLD1 and CTLD7 (52). Sera were assayed with ELISAs against these 3 domains; 23 sera reacted to just the CysR domain, 14 reacted to CysR and CTLD1, and 32 reacted to CysR, CTLD1, and CTLD7. The latter two groups were considered intramolecular epitope spreaders (beyond CysR). Median PLA2R titers were not significantly different among the 3 groups. Those reacting only to CysR were younger, had less proteinuria, more spontaneous remissions, and less progression. By multivariable analysis, both high PLA2R-titers and epitope spreading were independent risk factors for poor prognosis.

By contrast, Reinhard et al. analyzed 150 consecutive, immunosuppression-naïve, PLA2R-positive patients for epitope-recognition patterns and noted a fourth epitope (CTLD8) in addition to the CysR, CTLD1, and CTLD7 regions. All patients had antibodies targeting both N-terminal (CysR-FNII-CTLD1) and C-terminal (CTLD7-CTLD8) regions at study initiation. Total titers determined both degree of spreading and chance for remission, whereas spreading did not independently determine outcome.

### PLA2R antibodies: biomarkers or pathogenic?

Anti-PLA2R antibodies are indeed pathogenic and not merely biomarkers of activity. Meyer-Schwesinger et al. generated a transgenic mouse expressing murine PLA2R in podocytes which alone caused no injury (53). Upon exposure to rabbit anti-murine PLA2R-IgG, a lesion mimicking human MN was produced with proteinuria and hyperlipidemia. Injection of human anti-PLAR antibodies into these transgenic mice, however, did not produce disease, although sequence homology between human and murine PLA2R is only 72%. As minipigs express PLA2R in podocytes, Reinhard et al. administered plasma or purified IgG from patients with PLA2R-positive MN and produced disease mimicking early-stage MN with activation of complement but only low-level proteinuria (54).

Zahner et al. exposed transgenic *Rag2^-/-^* mice (cannot produce antibodies) expressing full-length human PLA2R in podocytes to purified IgG from PLA2R-positive patients. The result was proteinuria plus granular IgG-complement by immunofluorescence and subepithelial deposits with foot process effacement by EM. Eluted IgG from mouse glomeruli exclusively recognized PLA2R (55).

Indirect proof supporting the pathogenicity of PLA2R antibodies in humans comes from genetic analyses. Stanescu et al. performed a genome wide association study of 556 European PMN patients compared to 2, 338 racially matched controls and found significant single nucleotide polymorphisms (SNP) at 2 loci: PLA2R (SNP rs4664308, p=8.6x10^-29^) and, especially, HLA-DQA1 (SNP rs2187668, p=8.0x10^-93^) (56). Homozygosity for both at-risk SNPs gave an odds ratio for having PMN of 78.5. The relationship of these SNPs, as well as other SNPs at these 2 loci, was found in other populations, including South Asians (57), Spanish (58), Koreans (59), Taiwanese (60), and Han Chinese (61) and was confirmed in meta-analyses (62, 63). Lv et al. found 3 SNPs within PLA2R and 1 SNP in HLA-DQA1 were significantly associated with PMN in Han Chinese as well as with both positive PLA2R serology and glomerular PLA2R staining (~75% of patients with high-risk alleles at both loci had both antibodies and glomerular staining versus 0% if only low-risk alleles at both loci) (61).

The strongest association was with the HLA-DQA1 locus and not PLA2R, specifically with the intronic SNP rs2187668. Cui showed this intronic SNP was in linkage disequilibrium with specific HLA class II alleles (DRB1*1501 and DRB1*0301) that were independent risk factors for both PMN and circulating anti-PLA2R antibodies in Han Chinese (64). Specific amino acid substitutions within these alleles facilitated interactions with the epitopes of PLA2R. Controlling for DRB1*0301, the DQA1 intronic SNP rs2187668 lost significance. Two haplotypes containing these alleles were significantly associated with PMN (DRB1*1501-DQA1*0102-DQB1*0602 and DRB1*0301-DQA1*0501-DQB1*0201). Hence the intronic SNPs may merely be in linkage disequilibrium with the truly causative alleles/haplotypes. Xie et al. performed a GWAS of 3, 782 PMN patients and 9, 038 controls and confirmed the significant risk of both DRB1*1501 and DRB1*0301 in East Asians and DRB1*0301 and DQA1*0501 in Europeans (65).

Additional supporting evidence for the pathogenicity of PLA2R antibodies comes from studies addressing post-transplantation recurrence of PMN (66). Unlike *de novo* MN (usually secondary to infections or an alloimmune response) which occurs later (years), PMN can recur early in the first year or also later after years. Recurrence occurs in one-third (67, 68) to as much as one-half (69) (if protocol biopsies are routine). Whereas earlier, smaller studies did not find a significant relationship between PLA2R-positivity at the time of transplantation and recurrence (70, 71), more recent and larger studies have (67, 69, 72, 73). Among positive patients, higher titers increase recurrence risk with various ELISA cutoffs best fitting individual populations (45 RU/ml (72), 29 RU/ml (73), or 150 RU/ml (67)). Perhaps more important than baseline titers is their evolution over time post-transplantation. As in native kidneys, falling titers indicate immunologic remission and portend less recurrence. Persisting, rising, or reemerging titers increase the chance for relapse (70–72) and should prompt close clinical follow-up and protocol biopsy. Although some histologically recurrent cases may be benign (69), data indicate recurrent MN is usually progressive suggesting treatment sooner rather than later (e.g., with proteinuria ≥ 1 gram) (66).

Altogether, the evidence from animal models and indirectly from human investigations strongly supports that PLA2R antibodies are pathogenic and not epiphenomena. Titers remain as valuable targets for treating the individual patient and as surrogates for therapeutic trials.

### The antigenic-based classification of membranous nephropathy

Numerous antigenic targets besides PLA2R have been found in biopsy specimens of MN. Some antigens have been associated primarily with PMN, but most also have secondary causes less commonly. Others are uncommon causes of PMN but are predominantly associated with secondary causes. Typically, an antigen is identified by LM/MS or immunoprecipitation. Subsequently, the antigen can be detected in granular fashion along the glomerular capillary wall by immunohistochemistry or immunofluorescence. As confirmation, patient serum or IgG eluted from frozen kidney biopsy can be tested by WB against the antigen (74). Alternatively, the antigen can first be detected by WB of patient sera against human glomerular extract followed by immunoprecipitation and MS to identify the targeted protein (75).

Altogether, 15 such antigens have been described (**Table 2**) (9, 74). By far, PLA2R is most common, accounting for 70 – 80% of PMN cases. The others are all uncommon, and still about 10% of cases remain undefined. Some antigens have commercially available serologic assays for the corresponding antibodies. Some well-described associations exist between specific antigens and specific secondary causes. Importantly, one antigen does not equate to one secondary condition (9). Rather, a given antigen may be associated with several secondary causes, and a given secondary cause may be associated with multiple antigens.

**Table 2:** Defined antigenic targets for autoantibodies in primary membranous nephropathy.

Antigen	Location	Frequency*	Pathologic characteristics	Circulating antibodies	Clinical associations
PLA2R	Transmembrane	70-80%	IgG4 predominant Global granular staining Antigen detected by IHC, IF, and LM/MS (may be used for all of the antigens)	ELISA (quantitative) IIF (semiquantitative) commercially available Western blot most sensitive not commercial Titers correlate with disease activity	Infections (hep B and C, HIV, syphilis), sarcoidosis GVHD Malignancy (< 5%)
THSD7A	Transmembrane	~3%	IgG4 predominant Deposits scattered Antigen detected by IHC	ELISA (quantitative) IIF (semiquantitative) commercially available Western blot most sensitive not commercial Titers correlate with disease activity	Malignancy (25%)
NELL-1	Secreted	5-10%	IgG1 predominant May have segmental deposits on immunofluorescence Antigen detected by IHC	ELISA and Western blot No assays commercially available	Thiol-containing drugs Mercury containing TIMs Malignancy (up to 33%) NSAIDs Autoimmune diseases (up to 25%) GVHD (up to 20%)
Sema3B	Transmembrane and Secreted	~1% !0% of peds MN	IgG1 predominant TBM deposits Antigen detected by IHC	Western blot Not commercially available	Peds MN
HTRA1	Secreted	< 1%	IgG4 predominant Antigen detected by IHC	Western blot Not commercially available	None identified
NTNG1	Transmembrane	< 1%	IgG4 predominant Antigen detected by IHC	ELISA and Western blot Not commercially available	None identified
CNTN1	Transmembrane	≤ 2%	IgG4 predominant	IIF, commercially available	CIDP
NDNF	Secreted	1%	IgG1 predominant among IgG May have “full house” and C1q EM may show hump-like deposits	ELISA, commercially available	Syphilis
PCDH7	Transmembrane	~1%	No predominant subclass Lack of or weak C3 May have TRI	Only in-house assays None commercially available	Autoimmune disease Malignancy
FAT1	Transmembrane	~1%	IgG4 Mild C3	ELISA, Western blot Research purposes only None commercially available	Post-HSCT Associated with ABMR as *de novo* MN in the allograft
PCSK6	Secreted	2-5%	IgG4 or IgG1 predominant	ELISA, Western blot Research purposes only None commercially available	Heavy NSAID use
EXT1/2	Secreted	5%	IgG1 predominant “full house” and C1q typical Mesangial and subendothelial EDDs as well as subepithelial TRIs	None are available even for research	LMN (about 1/3) Prognosis of LMN better if EXT1/2 + versus -
NCAM1	Transmembrane	1%	No predominant subclass “full house” and C1q typical TRI	ELISA, IIF, Western blot For research purposes only Not commercially available	Autoimmune disease Proliferative lupus nephritis
TGFβR3	Transmembrane	< 1%	No predominant subclass “full house” in about 50%	None are available even for research	Lupus
MPO	Secreted	<1%	Unknown predominance Crescentic	ELISA readily available	MPO-positive vasculitis.

ABMR, antibody mediated rejection; EDD, electron dense deposits; ELISA, enzyme-linked immunosorbent assay; HSCT, hematopoietic stem cell transplant; IF, indirect immunofluorescence on tissue for antigen detection; IHC, immunohistochemical stain on tissue for antigen detection; IIF, indirect immunofluorescent serum antibody assay; LM/MS, laser microdissection/mass spectrometry; LMN, lupus membranous nephropathy; TBM, tubular basement membrane; TIM, traditional indigenous medication; TRI, tubular reticular inclusions.

*of all PMN patients, not restricted to only PLA2R-negative cases.

Thrombospondin type 1 domain-containing 7A (THSD7A), a transmembrane protein normally expressed in podocyte foot processes (76) functioning to stabilize the slit diaphragm (77), has been detected in 10% of PLA2R-negative cases (78). The predominant subclass is IgG4, and complement may be activated via the alternate pathway (3). The pathogenicity of these antibodies has been confirmed (76, 79). Up to 25% of THSD7A-positive cases may be secondary to malignancy (80). Both quantitative ELISA and IIF assays are commercially available (EUROIMMUN); titers correlate with disease activity (81).

Neural epidermal growth factor-like 1 (NELL-1), a secreted glycoprotein functioning in bone formation, was found to be the most common target antigen in PLA2R-negative cases, with geographic variability (e.g., 23% of North American PLA2R-negative patients (82), 5.9% of PLA2R-, THSD7A-double-negative French and Belgian PMN patients (82), 35% of Chinese PLA2R-, THSD7A-double-negative PMN patients (83), and 1.5% of all Japanese PMN patients (84)). Compared to PLA2R-positive MN, cases positive for NELL-1 tend to be older, although otherwise clinically similar. Biopsies differ in that segmental histochemical staining for NELL-1 is common and IgG1 is dominant or codominant (85). Circulating anti-NELL-1 antibodies have been detected using WB methodology (82, 83, 85), which is not widely available. Quantitative ELISA and an IIF assay were recently developed (86). Using tissue NELL-1 positivity as the gold standard, the ELISA had sensitivity/specificity of 61.3%/94.9% and the IIF 66.7%/98.6%, respectively. By adding WB, 83% of NELL-1-biopsy-postive patients were positive by at least one serologic method. The ELISA and IIF assays are not commercially available.

Multiple secondary causes of MN are associated with NELL-1 positivity. Malignancy is found in up to one-third (including lung, prostate, breast, kidney, skin, and others (85, 87)), coinciding with the increased age of onset of NELL-1 positive MN (85). Surgical cure of the malignancy produced MN remission (88). NELL-1 positivity mandates an aggressive search for malignancy, especially when over age 60. Use of sulfhydryl-containing medications such as lipoic acid (89), tiopronin (90), and bucillamine (91), as well as mercury containing traditional indigenous medications (92) and skin lightening creams (93) have been associated with NELL-1-positive MN, and remission has occurred with withdrawal of the agent. NELL-1 positive MN has also been associated with graft-versus-host disease (GVHD), autoimmune disease, and NSAID exposure (9).

Semaphorin 3B (Sema3B), a secreted protein, was recently identified in PLA2R-negative MN (94). Notably, ~75% of patients were in the pediatric age range, and affected adults were young (< age 40). IgG1 predominated. A WB correlated with disease activity. Sema3B-positive MN recurred after kidney transplantation (95). A commercial assay for circulating antibodies is not available.

Al-Rabadi et al. identified the secreted protein called high temperature requirement A serine peptidase 1 (HTRA1) as the target antigen of 3 – 4% of PLA2R-negative PMN from 2 large cohorts (75). Circulating antibodies detected by WB correlated with disease activity. Antibodies and tissue deposits were IgG4-predominant. No secondary cause(s) was apparent. The average age was 67 years. A commercial assay for circulating antibodies is not available.

Netrin G1 (NTNG1), a membrane-bound protein normally expressed in podocytes, was found to be the target antigen in 3/800 MN patients by NTNG1 and IgG4 staining using immunohistochemistry. Circulating IgG4 antibodies were detected by ELISA and WB. No secondary cause was detected (96). A commercial assay for circulating antibodies is not available.

Contactin 1 (CNTN1) is a membrane-bound protein expressed at paranodal regions in the peripheral nervous system. CNTN1 is also expressed in podocytes. Evaluating 1, 500 patients with chronic inflammatory demyelinating polyneuropathy (CIDP), Le Quintrec identified 11 with circulating IgG4 anti-CNTN1 antibodies of which 5 had biopsy proven MN (97). Immunofluorescence showed granular subepithelial IgG4-predominant deposits that colocalized by confocal microscopy with CNTN1. Immunohistochemical staining confirmed CNTN1. IgG4 eluted from frozen tissue reacted with CNTN1. Fehmi et al. identified 15 patients (mean age 59) with CNTN1-antibody positive neuropathy and MN; 4 of 295 MN patients without neuropathy were also serologically CNTN1 positive (98). Evaluation by both immunohistochemistry and LM/MS confirmed CNTN1. Overall, 75% responded with at least stabilization of neuropathy and nephropathy. An IIF assay for anti-CNTN1 antibodies is commercially available (EURIMMUN) (99).

Neuron-derived neurotrophic factor (NDNF) is a secreted protein that was shown to promote growth and migration of mouse neurons (100). Sethi et al. found NDNF as the target antigen in 8 cases of MN, including all 6 syphilis cases that were tested, 1 lung carcinoma and 1 HIV-positive patient (101). WB of eluted IgG from frozen biopsy tissues showed binding to NDNF. IgG1 was the predominant subclass, although IgG2 and 3 also were positive. Full-house positivity was found in 5/8 and C1q in 7/8 cases. Treatment of syphilis led to complete remission, as did rection of the lung carcinoma. By contrast, Wanderley et al. found 4 of 10 patients with syphilis-associated MN had PLA2R-positive staining, again IgG1 predominant, and all remitted with antibiotic therapy (102). An ELISA for NDNF antibodies is commercially available.

Protocadherins are a large family of transmembrane adhesion molecules predominantly expressed in the central nervous system and involved in neural development (103). Some members are expressed in the kidney, including podocytes and the slit diaphragm (104). At least 2 have been identified as target antigens in MN (protocadherin 7 and protocadherin FAT1).

Sethi et al. found protocadherin 7 (PCDH7) in 14 cases (6%) of PLA2R-negative MN (105). Circulating anti-PCDH7 antibodies were detected by WB. On biopsy, C3 was negative or minimally positive. There was no IgG subclass predominance. Six of the 14 had associated autoimmune or neoplastic disease, indicating both PMN and secondary causes are possible.

In the series of Sethi et al. no other antigens were co-expressed. By contrast, Machalitza et al. found circulating anti-PCDH7 antibodies by WB in 4 of 125 randomly selected serologically PLA2R-positive MN patients (3.2%) and 4 of 98 PLA2R-negative patients (4.1%) (106). Of the latter, 2 were NELL-1-antibody positive meaning 6/8 (75%) PCDH7-positive patients were also positive for a second antibody. PCDH7-positive glomerular staining by immunofluorescence was positive in 5 of 6 with available tissue along with the second antigen in all 6, and the clinical course mirrored the second antibody level, but not that of anti-PCDH7. Subsequently, they confirmed PCHD7-positivity by LM/MS in 3 of the original 6 cases but also in zero-hour transplant biopsies casting doubt on the pathogenic relevance of PCHD7 (107). No commercial assay for circulating PCDH7 antibodies is available.

Sethi et al. identified the protocadherin FAT1 as the target antigen in 14 patients with MN developing following allogeneic hematopoietic stem cell transplantation (HSCT) as a manifestation of chronic GVHD (108). IgG4 predominated and C3 staining was absent or minimal like with PCDH7. Protease digestion was required to detect FAT1 by immunohistochemistry. Rarely, PLA2R- (108) or NELL-1-positivity (109) may be found following HSCT instead of FAT1. Of note, FAT1 was also found to be the target antigen in 4 cases of *de novo* MN occurring post-kidney transplantation concurrently with antibody-mediated rejection (4 of 7 such cases); IgG4 was predominant (110). No commercial assay for circulating antibodies is available.

Proprotein convertase subtilisin/kexin type 6 (PCSK6), a secreted protein, was identified as a target antigen in 5 of 250 PLA2R-negative MN patients all of whom had prolonged heavy NSAID use (118). An additional 8 cases were detected in a validation cohort out of 118 quadruple negative (PLA2R, THDS7A, NELL-1, EXT2) patients of whom 5 were heavy NSAID users. Thus, 10/12 were NSAID associated. Five of 8 patients in the validation cohort had autoimmune diseases, including those without NSAID usage. IgG4 and IgG1 were codominant. Complete remission occurred in 8, of which 4 were only treated conservatively; partial remission occurred in 3 (2 conservatively treated). No commercial assay for circulating antibodies is available.

Rarely, patients with MN may have a rapidly progressive course with crescents on biopsy mediated by anti-GBM antibodies or by ANCA. Zou et al. studied 223 Chinese patients with ANCA-associated glomerulonephritis of whom 202 were anti-myeloperoxidase (MPO) positive; 27 (25 MPO-positive) had concurrent MN. Only 3/24 had detectable PLA2R antibodies and only 1/15 had PLA2R-positive glomeruli. IgG2 and IgG3 were dominant in glomeruli but MPO was not assessed in glomeruli. However, other groups have demonstrated glomerular MPO staining in cases of concurrent MPO-associated vasculitis and MN using immunoperoxidase (119) or double immunofluorescence and immunogold EM (120).

### Autoantigens in lupus membranous nephritis

Certain target antigens show a predilection for autoimmune disease-associated MN, especially lupus MN (LMN). The membrane-bound proteins exostosins 1 and 2 (EXT1/EXT2) are the target antigens in approximately 10% of PLA2R-negative PMN cases (111) and in a higher percentage of MN patients with autoimmune disease. About one-third of LMN cases may be EXT1/2 positive (112), with less severe kidney disease at presentation (112, 113), and with better prognosis than EXT1/2-negative LMN cases (112, 114). IgG1 is the predominant subclass, and “full-house” positivity (IgG, IgA, IgM) with positive C1q staining is common, found in over 70% (111). On EM, subepithelial deposits are invariably present, but mesangial deposits are typical and subendothelial deposits may be found. Most are young women with a mean age of 35. Some patients have diagnosable autoimmune disease (lupus, Sjogren’s syndrome, mixed connective tissue disease), some just autoantibody positivity, whereas others have neither, suggesting PMN (115). A serologic assay for anti-EXT1/2 antibodies has not been developed, and diagnosis requires demonstration of tissue expression.

Similarly, membrane-bound neural cell adhesion molecule 1 (NCAM1) was found in 6.6% of LMN cases and 2% of PMN cases (116). On immunofluorescence microscopy, 40% had “full house” positivity and 55% were C1q-positive. There was no consistent IgG subclass predominance. Most patients were young females (average age 34). Forty percent of the 18 NCAM1-positive LMN patients had neuropsychiatric manifestations (cerebritis, seizures, psychosis), unlike EXT1/2-positive LMN cases where these are rare. Circulating antibodies were detectable by WB and IIF, although they are not commercially available.

A third target antigen, TGF-β receptor 3 (TGFBR3), was found in association with LMN. Caza et al. identified 17 TGFBR3-positive MN patients, including 11 of 199 consecutive LMN cases (6%) (117). Altogether, 16 of the 17 TGFBR3-positive MN cases had lupus. “Full house” staining occurred in 47%. No consistent IgG subclass predominated. TGFBR3 and IgG colocalized in glomerular immune deposits. Like EXT1/2, circulating antibodies could not be detected by Western blotting, IIF, or immunoprecipitation.

There are no data available to support altering overall rheumatologic management of systemic lupus based on positive EXT1/EXT2, NCAM1, or TGFBR3 staining in LMN. To our knowledge, positivity for any of the three antigens is not predictive of future lupus flares. However, from the kidney perspective, there may be prognostic implications; as noted above, EXT1/EXT2-positive LMN cases fare better than negative ones (118–120).

### Dual antigen positivity

A minority of MN cases involves 2 antigens (see **Table 3**). As noted above, dual positivity of PCDH7 with a second antigen was common in one study (6/8 or 75%), including 4 PLA2R- and 2 NELL-1 cases; the clinical course mirrored the titer of the later antibodies, not anti-PCDH7 (106).

**Table 3:** Dual antigen positivity.

Author/year	Antigens	Number/frequency	Clinical features	Comments
Larsen/2015 (121)	PLA2R and THSD7A	2 cases, 1%	N/A	100% correlation with serologic analysis for both antigens in both patients
Wang/2017 (122)	PLA2R and THSD7A	2 cases/<1%	N/A	100% correlation with serologic analysis for both antigens in both patients
Zaghrini/2018 (81)	PLA2R and THSD7A	8 cases*/16% of THSD7A-positive patients	No difference in age, sex, baseline clinical values, or malignancy association versus THSD7A single positive patients	Using quantitative ELISA, variable titers of each antibody, some negative
Pan/2023 (123)	PLA2R and THSD7A	6 cases/3%	No clinical differences from single antigen positive patients	All 6 negative for circulating anti-THSD7A antibodies
Yang/2024 (124)	PLA2R and THSD7A PLA2R and Nell-1	3 cases/<1% 3 cases/<1%	Only 3 of 6 presented with nephrotic syndrome More likely IgG1 and longer time to remission**	4 of 6 PLA2R-positive serologically. All THSD7A- and NELL-1-positive patients negative serologically
Machalitza/2025 (106)	PLA2R and THSD7A PCDH7 and PLA2R PCDH7 and NELL-1	1 case/<1% 4 cases/3.2% of PLA2R positives 2 cases/10.5% of NELL-1 positives	Clinical course aligned with PLA2R or NELL-1 titers, not with PCDH7	6 of 8 PCDH7-positives were dual positive
Nasr/2026 (125)	PLA2R and THSD7A PLA2R and NELL-1 PLA2R and PCDH7 NCAM1 and EXT1/2 NELL-1 and CNTN1 NDNF and NELL-1 CNTN1 and PCDH7	1 case/frequencies not available 1 case 1 case 2 cases 2 cases 1 case 1 case	8 of 9 had nephrotic syndrome 3 of 9 eGFR < 60 ml/min/1.73 m^2^	4 cases from Mayo clinic, 5 from Arkana Laboratories all evaluated by liquid chromatography followed by mass spectrometry

*these 8 include the 4 cases of Larsen (2) and Wang (2).

**includes additional 11 cases obtained from literature review all compared to consecutive cases from their center.

Dual positivity for PLA2R and THSD7A is found in a small percentage. Larsen et al. found that 2 of 258 MN biopsies (~1%) had dual positive glomerular staining with corresponding circulating autoantibodies (121). Wang et al. found 2 dual positive patients, both tissue antigen positive and circulating antibody positive, among 578 Chinese PMN patients (122). Among 49 THSD7A-positive patients from multiple cohorts, Zaghrini et al. found 8 dual-positive patients (including the 4 described by Larsen et al. and Wang et al.). Three of the other 4 identified cases resulted from screening 1, 012 MN patients (0.3%) (81). Comparing titers of each antibody, either antibody could predominate, or titers could be roughly equal. Of 194 Chinese PMN cases, Pan et al. found 6 (~3%) dual positive for PLA2R and THSD7A by immunofluorescence microscopy without clinical differences from single antigen-positive patients; all 6 were negative for circulating anti-THSD antibodies (123).

Yang et al. performed immunohistochemical staining for PLA2R, THSD7A, and NELL-1 on 827 Chinese patients and identified 6 (0.7%) with dual positivity, including 3 with PLA2R- and THSD7A-positivity and 3 with PLA2R- and NELL-1-positivity (124). PLA2R was serologically positive in only 4 of the 6 and all THSD7A-tissue-positive cases were serologically negative for THSD7A antibodies, as were all Nell-1-postive patients for NELL-1 antibodies. They also performed a systematic literature review identifying 43 additional dual antigen positive patients, of which 11 provided detailed information. Comparing these 17 total patients to 141 consecutive PLA2R-positive patients from their center, they found only a higher percentage of IgG1-positivity and a longer time to achieve remission in dual positive patients.

Nasr et al. performed LM/MS on 155 PLA2R-negative MN patients and found 4 (2.6%) dual antigen-positive (NCAM1+EXT1/2, NELL-1+NDNF, and 2 cases of NELL-1+CNTN1), all confirmed by immunohistochemistry (125). In this study, five additional dual antigen-positive cases from Arkana Laboratories detected by immunohistochemistry/immunofluorescence were confirmed by LM/MS (NCAM1+EXT1/2, PLA2R+NELL-1, PLA2R+THSD7A, PLA2R+PCDH7, and CNTN1+PCDH7). Although both antigens were considered positive, the spectral counts of one generally predominated. All 9 had nephrotic range proteinuria, and 8 had hematuria. IgG1 predominated in 3/5 tested, IgG4 in 2/5. In the majority, a secondary cause associated with one of the antigens was present. Since LM/MS and/or immunohistochemistry for other antigens are not routinely performed on PLA2R-positive cases, dual positivity is probably more common than data suggest.

### Intracellular antigens

Antibodies may develop in MN patients against intracellular podocyte antigens representing intermolecular epitope spreading. The 3 most notable ones are α-enolase, aldose reductase, and superoxide dismutase (126, 127). Approximately two-thirds of both PLA2R-positive and PLA2R-negative patients have these antibodies (128). In one study, antibodies to superoxide dismutase and α-enolase independently associated with reduced remission and lower GFR during follow-up (128). Interestingly, using LM/MS, there was no difference in the high spectral counts for α-enolase between single antigen-positive, dual antigen-positive, nor non-MN cases. High spectral counts for neither aldose reductase nor superoxide dismutase were detected by LM/MS in single or dual antigen-positive cases (125).

### Treatment

**Figure 1:**
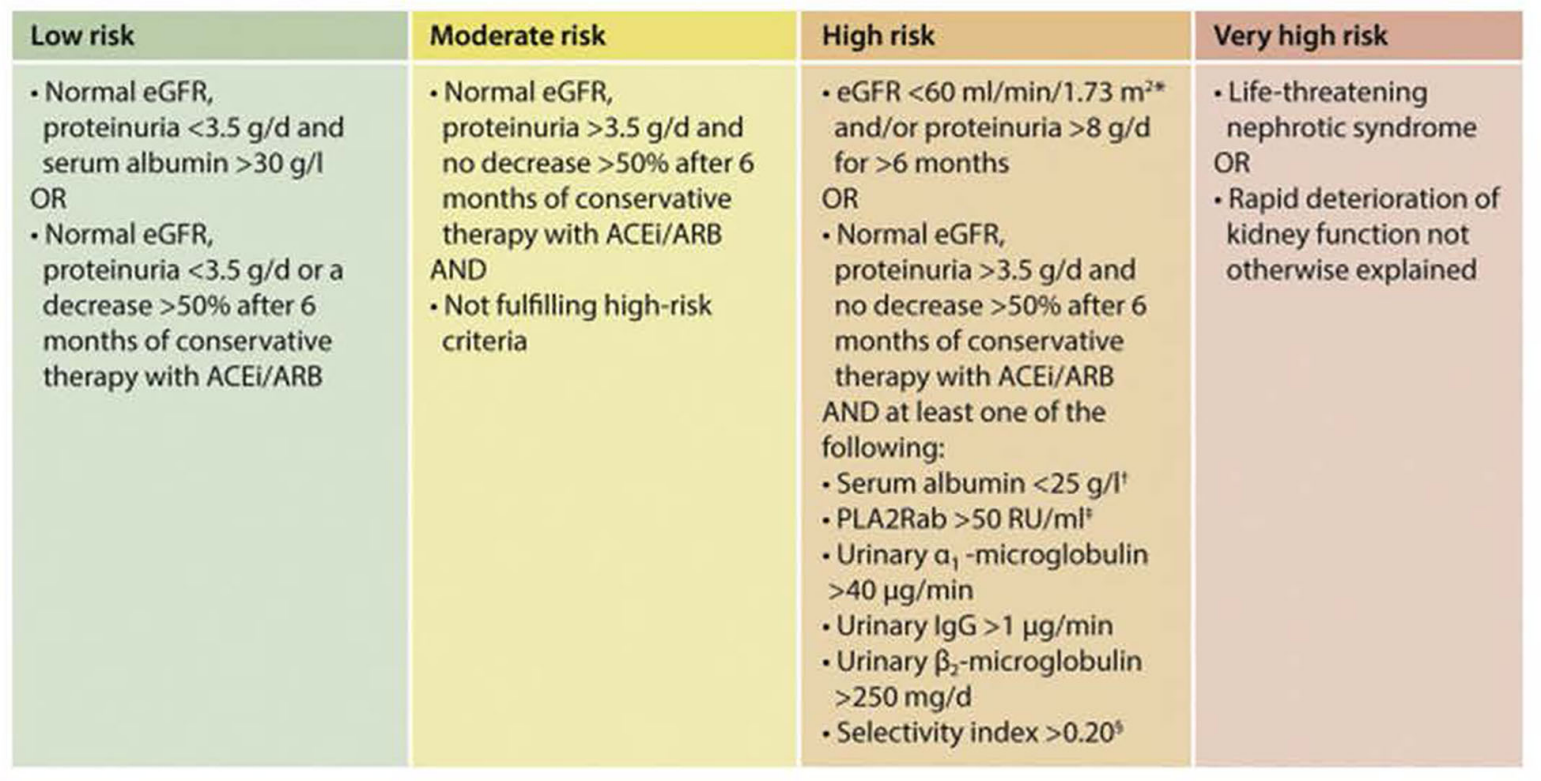
Clinical criteria for assessing risk of progressive loss of kidney function. eGFR and PCR are used in routine clinical care. Other biomarkers may not be available in all centers; this table provides an overview of useful biomarkers. ^∗^Most studies have used serum creatinine (SCr) values to guide management, and SCr values >1.5 mg/dl (133 μmol/l) are often used to define kidney insufficiency. An eGFR value of 60 ml/min per 1.73 m^2^ defines kidney insufficiency in a young adult. It is important to realize that eGFR decreases with age, and an SCr value of 1.5 mg/dl (133 μmol/l) reflects an eGFR of 50 ml/min per 1.73 m^2^ in a 60-year-old male patient and 37 ml/min per 1.73 m^2^ in a 60-year-old female patient. Thus, when using eGFR in risk estimation, age should be taken into account. ^†^Serum albumin should be measured by BCP or immunometric assay. ^‡^Cutoff values are not validated. Anti-PLA2R antibodies should be measured at 3-to-6-month intervals, the shorter interval being performed in patients with high anti-PLA2R antibodies levels at baseline. Changes in anti-PLA2R antibodies levels during follow-up likely add to risk estimation. Disappearance of anti-PLA2R antibodies precedes clinical remission and should lead to refraining from additional therapy. Detailed data are lacking. ^§^Selectivity index is calculated as clearance of IgG/clearance of albumin. ACEi, angiotensin-converting enzyme inhibitor; ARB, angiotensin II receptor blocker; BCP, bromocresol purple; eGFR, estimated glomerular filtration rate; IgG, immunoglobulin G; PLA2Rab, antibodies against the M-type phospholipase A2 receptor. Copied from KDIGO Guidelines, Brad H. Rovin, Sharon G. Adler, Jonathan Barratt, Frank Bridoux, Kelly A. Burdge, Tak Mao Chan, H. Terence Cook, Fernando C. Fervenza, Keisha L. Gibson, Richard J. Glassock, David R.W. Jayne, Vivekanand Jha, Adrian Liew, Zhi-Hong Liu, Juan Manuel Mejía-Vilet et al. *Kidney Int*, 100 (2021), pp. S1-S276.

All patients with MN should have optimized supportive care, including strict BP control, RAS inhibition, salt restriction, moderated protein intake, SGLT2 inhibition, smoking cessation, weight loss if obese, and regular exercise. Unless eGFR has declined or severe complications have developed (very high risk, requires immediate immunosuppression), patients can be followed for up to 6-months for possible spontaneous remission (SR). The KDIGO 21 Guidelines for the treatment of Glomerular Disease (46) state the following: the decision to immunosuppress depends on risk of kidney disease progression (**Figure 1**). Low risk patients can be followed expectantly; moderate risk patients should receive immunosuppression (rituximab or calcineurin inhibitors); high risk patients should receive rituximab, cyclophosphamide/glucocorticoids, or calcineurin inhibitors/rituximab; and very high-risk patients should receive cyclophosphamide/glucocorticoids (46).

Studies from several decades ago (see **Table 3**) provided evidence for alkylating agent-based therapy on long-term reduction of hard end-points (ESKD and death) (129–134). More recently, the UK RCT evaluated patients with declining kidney function, defined as ≥ 20% eGFR decline over 2 years comparing steroids/chlorambucil, cyclosporin, and supportive therapy (135). The primary endpoint, a further 20% decline, occurred in 58% given corticosteroids/chlorambucil versus 84% given supportive treatment (p=0.0042); cyclosporin (81% reached the primary endpoint) was no different than supportive treatment. Although initial studies of corticosteroids/alkylating agents used chlorambucil (131, 132, 136), cyclophosphamide was shown to be equally effective (133, 134) with less toxicity and is now the preferred alkylating agent for very high-risk patients.

Over the past 5–10 years, however, much research has centered on rituximab and other B-cell depleting agents, due to ease of administration and the adverse side-effect profile of steroids/alkylating agents. We discuss in detail observational studies using rituximab followed by the RCTs (**Table 3**). We will also discuss the approach to rituximab resistance that occurs in 20 – 40% of treated patients.

Remuzzi et al. first reported use of rituximab (375 mg/m^2^ weekly x 4 doses, the typical protocol for treating lymphoma) in 8 patients with PMN finding a significant reduction in proteinuria and improved serum albumin (137, 138). Fervenza et al. gave 1 gram rituximab twice (2 weeks apart, the typical protocol for rheumatoid arthritis) to 15 nephrotic PMN patients with 10 receiving a second course at 6 months for persisting proteinuria and B-cell repopulation (139). Proteinuria was reduced about 50% at 12 months, and 8 patients had remission. B-cell reconstitution began at 3 months with most having normal counts at 6 months. By contrast, in patients with autoimmune diseases, reconstitution typically begins at 6 months and takes a year to complete. Neither rituximab levels at any time point nor development of anti-rituximab antibodies (ARA) in 6 patients correlated with response.

Fervenza et al. gave 20 patients the lymphoma protocol with 2-year follow-up and obtained similar post- and pre-dose levels as the rheumatoid arthritis protocol. Both regimens gave significantly lower rituximab levels compared to similarly dosed rheumatoid arthritis patients (140). B-cell depletion was greater with the lymphoma protocol, but both had faster B-cell recovery than vasculitis patients given the rheumatoid arthritis protocol. Rituximab levels did not correlate with response. At 2 years, 16/18 had remission, including 4 complete. The lymphoma protocol was effective in 13 calcineurin inhibitor dependent patients (11 concurrently treated with MMF); all 13 were in remission at 12 and 30 months, including 3 that were retreated after relapse (141). It appears that the rheumatoid arthritis protocol and the lymphoma protocol are roughly equivalent and both are considered high-dose initial therapy (≥ 2 grams total). However, rituximab levels are lower and B-cell repopulation occurs sooner than similar regimens given to non-nephrotic patients, presumably from urinary loss of rituximab.

In the largest study to date, Vargas-Brochero retrospectively analyzed 159 patients treated with rituximab over a 23-year period at the Mayo Clinic; 75.5% were PLA2R-positive (142). Over half (84 or 52.8%) were previously immunosuppressed, including 45 non-responders and 39 relapsers. Most received the rheumatoid arthritis protocol (80%) or the lymphoma protocol (18%). Seventy-three percent were retreated, usually at 6 months, with an overall median dose of 4 grams. In the 85 patients with 5 years follow-up, proteinuria decreased from 8.75 g/day to 0.50 g/day and eGFR increased from 54.56 to 67.43. At last follow-up, 88.1% had a response, including 66 complete remissions, 77 partial remissions, and 2 proteinuria reductions > 50%. The median time to partial remission was 6.8 months and to complete remission was 22.6 months. Among 81 PLA2R-positive patients with serial monitoring, the median time to immunologic remission was 4.9 months, well ahead of clinical remissions. Including patients treated with additional therapy, 14 eventually reached ESKD after 67 months (median).

Huang et al. systematically reviewed the English literature regarding the efficacy of rituximab and identified 21 studies involving 602 patients (143). The overall remission rate was 67%, including a complete remission rate of 26%. Proteinuria was reduced 62% (P<0.00001). There was no difference in remission rates or proteinuria reduction based on percentage of patients with prior immunosuppression or percentage of PLA2R-positivity.

The preferred dose of rituximab remains uncertain. The long-term results of Vargas-Brochero (142) (median dose 4 grams) support high-doses (> 2 grams). Others studied lower doses. Although initially using the lymphoma protocol, a multicenter Italian group switched to a regimen of one 375 mg/m^2^ dose followed by a second dose only if B-cells remained > 5 cells/mm^3^ (144). Subsequently, Cravedi et al. (145) compared this titrated rituximab regimen in 12 patients (11/12 only needed 1 dose) to 24 historical controls given the lymphoma protocol. There was no difference either in proteinuria decline or serum albumin increase with similar proportions achieving remission at 1 year. Mean total rituximab exposure was 750.8 versus 2786.7 mgs with estimated cost savings of $13, 000 per patient. The lower dose regimen appeared similarly effective in previously immunosuppressed patients.

Others also found reasonable response rates with lower dose initial regimens (< 2 grams total). Bagchi et al. gave two 500 mg doses 1 week apart to 21 immunosuppression-resistant cases and achieved 62% remission (146). Michel et al. found a response rate of 82.6% with no difference between 11 patients given only 2 weekly doses of 375 mg/m^2^ and 17 given either 3 or 4 weekly doses or the rheumatoid arthritis protocol (147). Wang et al. treated 36 non-responsive patients with 1 (3 patients), 2 (11 patients), 3 (7 patients), or 4 (15 patients) 375 mg/m^2^doses (148). Overall, 15/36 (41.7%) responded. There was no difference in total rituximab dose between responders and non-responders.

By contrast, Moroni et al. gave 1 (18 patients) or 2 (16 patients) doses of rituximab (375 mg/m^2^) to 34 IMN patients and found that at 12 months only 15 responded (5 complete, 10 partial) and 19 had no response (149). There was no difference between those receiving 1 or 2 doses or between treatment naïve patients or those previously immunosuppressed.

Seitz-Polski et al. compared a lower dose regimen (375 mg/m^2^, 2 doses, 1 week apart) with the rheumatoid arthritis protocol and found the higher dose regimen had higher rituximab levels and lower CD19+ cells at 3 months, as well as lower PLA2R1 antibodies at 6 months, again suggesting reduced response with lower doses. Remissions were significantly higher at 6-months with the higher dose regimen (64% versus 30%, p=0.02) but not before treatment modification by last follow-up (86% versus 67%, p=0.12) (150). Others found reduced response rates with lower dose regimens (151). Ales Rigler et al. obtained only a 38% remission rate at 12 months after 29 rituximab treatments (26 patients), 21 of which received 375 mg/m^2^ once (152). In our opinion, a lower dose regimen should only be considered in those at moderate risk of progression.

The impressive benefits of rituximab demonstrated in observational studies are tempered by the significant percentage of SRs occurring with conservative therapy, even with high level proteinuria. A study followed 328 nephrotic PMN patients treated conservatively at multiple Spanish centers (153). Overall, 104 (31.7%) developed a SR, 52 of which proceeded to complete remission, including 26% SRs among patients with 8–12 g/day proteinuria (11 partial and 13 complete) and 21.5% among those with > 12 g/day (4 partial and 7 complete). After 91 months average follow-up in those with SR, no patient required immunosuppression, none progressed to ESKD, and only 2 died, compared to 78.5%, 18.7%, and 24 deaths, respectively, for those without SR. Only 5.7% (6/104) with SR relapsed. Similarly, Hoxha et al. reported remission in 10 of 11 conservatively treated antibody-negative patients with 9 g/day proteinuria and serum albumen 2.8 mg/dl at baseline (154).

Several RCTs support the efficacy of rituximab (see **Table 4**). The GEMRITUX study group randomized 77 French patients with persisting nephrotic syndrome after 6 months of non-immunosuppressive antiproteinuric treatment (NIAT) to continued NIAT alone or NIAT combined with low-dose rituximab (375 mg/m^2^ given twice, 8 days apart) with median follow-up of 17 months (155). The primary endpoint, partial or complete remission at 6 months, was non-significant (35.1% with rituximab versus 21.1% control), although a *post-hoc* composite endpoint of > 50% proteinuria reduction and > 30% increase in serum albumin was significant (40.5% with rituximab versus 13.2% control, p<0.01). In the PLA2R-positive subset (~70%), levels were significantly reduced at 3 and 6 months with rituximab but only at 6 months without rituximab. Complete immunologic remission was significantly greater with rituximab at both 3 (56% versus 4% p < 0.001) and 6 months (50% versus 12%, p=0.004). During extended follow-up (median 17 months), those randomized to rituximab had significantly lower proteinuria, higher serum albumin, and remission prior to any immunosuppression modification (64.9% versus 34.2%, p<0.01). There were no safety concerns.

**Table 4:** Notable randomized controlled trials treating primary membranous nephropathy.

Study/year	Number	Follow-up	Regimen	Control	1° Endpoint	Result	Comments
Ponticelli/ 1989 (131, 132)	81	10 years	Steroids/chlorambucil alternating monthly for 6 months	Supportive therapy	ESKD rate Remission rate Time not nephrotic	8% vs 40%, p=0.0038 83% vs 38%, p=0.0000 58% vs 32%, p=0.0001	Clear long-term hard endpoint benefit at 10 years
Ponticelli/ 1992 (136)	92	54 months	Steroids/chlorambucil alternating monthly for 6 months	Steroids alone, at equivalent dosing to steroids in alternating regimen	Time (%) free from nephrosis at 1, 2 3, 4 years	58, 54, 66, 62% versus 26, 32, 40, 42% P=0.002, 0.029, 0.011, 0.102	Effect most significant at 1 year, diminished over time, NS at year 4
Ponticelli/ 1998 (133)	95	36 months 42 months	Steroids/chlorambucil alternating monthly for 6 months	Steroids/cyclophosphamide alternating monthly for 6 months	Remission rate Relapse rate Time (%) free from nephrosis	82% vs 93%, p=0.116 30.5% vs 25% 58.6% vs 2.5%, p=0.534	Equally effective GFR no different Cyclophosphamide appeared safer
Jha/ 2007 (134)	93	10 years	Steroids/cyclophosphamide alternating monthly for 6 months	Supportive therapy	Remission rate Dialysis free at 10 years ESKD/2x creat/death	72% vs 35%, p<0.0001 89% vs 65%, p=0.016 21% vs 56%, p=0.0006	Proteinuria lower, GFR higher with treatment
UK RCT/ 2013 (135)	108	3 years	Steroids/chlorambucil alternating monthly for 6 months *or* cyclosporin	Supportive therapy	≥ 20% decline in excretory function (CG equation)	58% vs 84%, p=0.0042 81% vs 84%, p=NS	Required ≥ 20% decline in function in 2 years prior Cyclosporin no benefit
GEMRITUX/ 2017 (155)	75	17 months	Rituximab 375 mg/m^2^ x2–1 week apart plus NIAT	NIAT alone (non-immunosuppressive antiproteinuric therapy)	6-month remission	35% vs 21%, p=0.21	Greater anti-PLA2R depletion with rituximab 65% vs 34% had remission on extended follow-up, p<0.01
MENTOR/ 2019 (156)	130	24 months	Rituximab 1 g x2, 14 days apart	cyclosporin	Remission at 24 months	60% versus 20%, p<0.001	Rituximab superior Decline anti-PLA2R faster and greater with rituximab
RI-CYCLO/ 2021 (157)	74	12 months	Rituximab 1 g x2, 14 days apart	Steroids/cyclophosphamide alternating monthly for 6 months	Complete remission at 12 months	16% vs 32%, NS. Total remission: 62% vs 73%, NS at 12 months and 83 vs 82% at 24 months	Pilot trial, no difference in benefit or harm with rituximab
STARMEN/ 2021 (158)`	86	24 months	Steroids/cyclophosphamide alternating monthly for 6 months	Tacrolimus full-dose x 6 months then 3-month taper plus rituximab 1 g at 6 months	Remission at 24 months	83.7% vs 58% (RR 1.44, 95% CI 1.08 – 1.92)	Significantly higher PLA2R response with cyclical regimen at 3- and 6-months
Brglez/ 2025 (159)	64	24 months	Personalized approach based on epitope spreading: At baseline: NIAT in non-spreaders x 6 months High-dose rituximab (1g x2) in spreaders At 6 months if no Remission	GEMRITUX protocol: At baseline for all: 6-months NIAT At 6 months: Rituximab 375 mg/m^2^ x2–1 week apart if no remission	Remission at 12 months	67% vs 35%, p=0.01	In 32 baseline spreaders, remission in 15/17 vs 4/15, P=0.0008 4 patients in each group did not need rituximab

NS, non-significant; RR, relative risk; UK RCT, United Kingdom randomized controlled trial.

The MENTOR Investigators randomized 130 North American patients to high-dose rituximab (the rheumatoid arthritis protocol) or cyclosporin (156). Failure to decrease proteinuria by 25% at 6-months was considered treatment failure. Otherwise, rituximab was re-dosed at 6 months, unless complete remission, and cyclosporin continued for another 6 months also unless complete remission. The primary endpoint, remission at 24-months, occurred in 39 patients (60%) in the rituximab group versus 13 (20%) with cyclosporin (p<0.001 for both non-inferiority and superiority). The effect was consistent regardless of baseline PLA2R-antibody status (~75% positive in each group). At 24 months, 35% of rituximab-treated patients had a complete remission versus none given cyclosporin. Among PLA2R-positive patients, the reduction in antibody levels was faster and greater with rituximab.

The RI-CYCLO Investigators randomized 74 patients to either high-dose rituximab (the rheumatoid arthritis protocol) or a 6-month cyclical regimen of steroids/cyclophosphamide with a primary endpoint of complete remission at 12 months in a multi-center pilot trial (157). At 12 months, 6 (16%) rituximab patients versus 12 (32%) steroid-cyclophosphamide patients achieved complete remission (OR, 0.4, 95% CI, 0.13 – 1.23), with 62% versus 73%, respectively, achieving complete or partial remission. By 24 months, the rates of complete remission or any remission were nearly identical (42% versus 43% and 83% versus 82% for rituximab versus steroids-cyclophosphamide, respectively). Serious adverse events were similar. Anti-PLA2R antibodies, present in 66% of those tested, declined in both arms, although faster with rituximab.

The STARMEN Investigators randomized 86 patients into a 6-month cyclical regimen of steroids/cyclophosphamide versus full dose tacrolimus for 6 months followed by a 3-month taper plus rituximab 1 gram given once at 6 months with the primary endpoint of any remission at 24 months (158). The corticosteroid/cyclophosphamide regimen had a significantly higher remission rate (83.7% versus 58.1%), including significantly more complete remissions (60% versus 26%), as well as a significantly higher immunologic response at 3 and 6 months in PLA2R-positive patients. Adverse events were similar.

A multi-center French RCT utilized epitope spreading to allow for personalized therapy with rituximab. Brglez et al. randomized 64 PLA2R-positive PMN patients to the GEMRITUX protocol (31 patients, 6 months of symptomatic treatment followed by two 375 mg/m^2^ doses 1 week apart if no remission) or personalized therapy based on epitope spreading at baseline and 6-months (33 patients): at initiation, non-spreaders (16 patients) were treated with the GEMRITUX protocol (6 months of symptomatic treatment followed by rituximab if no remission and still non-spreading) (159). However, if spreading developed by 6 months, high-dose rituximab (rheumatoid arthritis protocol) was given instead of the GEMRITUX protocol. Those with spreading at initiation (17 patients) were treated immediately with high-dose rituximab; if no remission at 6 months and still spreading, high-dose rituximab was repeated, and if no remission but no longer spreading at 6 months, low-dose rituximab was given (375 mg/m^2^ x 2 doses). At 12 months the remission rate was significantly higher with personalized therapy (67% versus 35%, p=0.01) as was improved eGFR (p=0.0498). Multivariable analysis showed that 12-month remission was associated with delay before the first infusion, not the cumulative dose (median 1, 350 mgs personalized versus 2, 000 mgs). There was no difference in rate of spontaneous remissions suggesting the personalized group was not overtreated.

Rituximab has been combined with lower dose steroids/cyclophosphamide regimens in non-RCTs. Zonozi et al. treated 60 consecutive PMN patients with rituximab (rheumatoid arthritis protocol initially, with subsequent 1-gram doses repeated every 4 months until month 24) combined with both oral cyclophosphamide for 8 weeks and prednisone tapered to 0 over 28 weeks with a 38-month median follow-up (160). They obtained 100% partial remission (median 3.4 months) and complete remission in 83% by 2 years. Immunologic remission of PLA2R-positive patients (< 14 RU/ml) occurred in 86% at 3-months and 100% at 6-months. Relapses occurred in 10%, 2 years after B-cell reconstitution. No significant safety issues emerged.

Vink et al. combined rituximab (Rheumatoid arthritis protocol), oral cyclophosphamide for 8 weeks and steroids (two 1-gram iv boluses followed by 3 weeks oral prednisone) in 26 PLA2R-positive PMN patients with a median 26-week follow-up (161). Immunologic remission (< 14 RU/ml) by 8 weeks occurred in 88% (73% < 2 RU/ml). Remission occurred in 21, but 2 relapsed and 5 did not respond. These 7 required further treatment (5 with 2 rituximab doses, 1 with 2 rituximab doses plus bolus steroids, and 1 with tacrolimus) and all had remission. Thus, they achieved 100% remission with the majority (19/26) receiving less global immunosuppression. The decision for repeated immunosuppression was based solely on clinical grounds; however, 5 of 7 needing retreatment were identifiable by an estimated PLA2R-antibody t_1/2_ > 7days at 8 weeks. Four had serious adverse events.

### Rituximab resistance

About 20 – 40% of rituximab-treated patients are resistant potentially resulting from altered rituximab bioavailability, anti-rituximab antibodies (ARA), and/or chronic scarring in the absence of immunologic activity.

Reduced bioavailability leads to undertreatment, especially with low-dose regimens. Rituximab binds to albumin and may be lost in the urine (162), especially with severe nephrosis. Serum levels are lower with nephrosis compared to similar dosing in autoimmune diseases such as myasthenia (163) or rheumatoid arthritis (140). An ELISA (LISA-TRACKER Duo Rituximab, EUROIMMUN) can quantitate free rituximab levels. Teisseyre et al. found that 38 of 68 patients had undetectable levels (< 2µg/ml) 3 months after the last infusion and had a significantly lower chance of remission at 6 months (32% versus 80%, p<0.0001) and 12 months (42% versus 83%, p=0.001) compared to those with > 2 µg/ml, results that remained significant by multivariable analysis (164). Only baseline serum albumin independently predicted 3-month rituximab levels with the optimal threshold of 2.25 g/dl, presumably a proxy for the severity of nephrosis.

Allinovi et al. modified the serum LISA-TRACKER Rituximab ELISA for use in urine and studied 25 Italian PMN patients (165). Applying to urine the manufacturer-defined cut-off for serum rituximab (≥ 2 µg/ml), 14/25 had detectable urinary rituximab immediately after the second of two 1-g doses, and 11 were considered undetectable. Note that urine creatinine was not measured, and urinary concentration was not accounted for. By ROC curve analysis, a cut-off of 1.7 µg/ml for urinary rituximab was optimal for predicting remission (complete or partial) with sensitivity of 100% and specificity of 64.7% (AUC = 0.838, p<0.01). PLA2R-depletion was significantly more frequent with urinary levels < 2 µg/ml (80% versus 17%, p=0.008) suggesting enhanced bioavailability. Retreatment of 8 non-responsive patients (all with urinary levels ≥ 2 µg/ml) produced remission in 5.

Other factors besides urinary loss may determine rituximab bioavailability. Rituximab may be internalized and degraded as a tripartite complex of rituximab-CD20-Fc gamma receptor IIb (FcγRIIb) reducing antibody-dependent-cellular-cytotoxicity (ADCC), a key mechanism for rituximab to deplete B-cells. B-cell malignancies with higher expression of FcγIIb, even within the same tumor type, are more resistant to rituximab (166). In systemic lupus, variability in response to rituximab *in vitro* was mediated by the degree of FcγRIIb expression, which may explain the reduced clinical response to rituximab in such cases (167). The differential expression of FcγRIIb on B-cells in PMN has not been evaluated as a factor explaining rituximab bioavailability or resistance. Additionally, IgG antibodies such as rituximab bind to the neonatal Fc receptor (FcRn) which protects them from lysosomal degradation and prolongs their half-life (168). Differential expression or function of FcRn reducing rituximab bioavailability in MN have not been explored.

Rituximab resistance may result from ARA which also may be assessed by a commercially available ELISA (LISA-TRACKER Duo Rituximab, Theradiag Croissy-Beaubourg, France) with a cutoff for positivity of 5 ng/ml of free (unbound) antibody (169). Other methodology has been used, such as an electrochemiluminescent assay (170). ARA result in lower serum rituximab levels and impair B-cell depletion (171), and they can neutralize the cytotoxic potential of rituximab in *in vitro* assays (170). The frequency with which ARA develops depends largely on the disease being treated. ARA are rare with treatment of lymphoma (172). Patients with systemic lupus have much higher rates of ARA formation (173) compared to those with ANCA-associated vasculitis (173) or rheumatoid arthritis (174).

Approximately one-quarter to one-half of rituximab treated MN patients develop ARA (169, 175, 176). In rituximab-naïve patients, the median interval to ARA development is 9 months (176). They are not detectable at 3-months in naive patients but can be found at 6-months (169). Some studies indicate no difference in 6-month remission rate with ARA development detected at that time (139, 169); however, subsequent relapse rates are significantly higher (169), and ARA may necessitate a second course of therapy. In MN, the rate of ARA positivity progressively rises with subsequent courses (176), like in rheumatologic diseases (170). Risk factors for developing ARA in MN include higher body weight, higher anti-PLA2R antibody titer, higher IL-17A levels after *in vitro* stimulation, and lower 25-hydroxyvitamin D levels (177).

A retrospective analysis of 74 rituximab-treated PMN cases (57 rituximab-naïve) found ARA in 35 (47%). Among 12 non-naïve patients with available baseline assays, 9 had preexisting ARA (176). Naïve patients rarely had antibodies detected before 6-months, whereas 40% of previously treated patients had ARA detectable in less than 3 months. Notably, all non-naïve patients with pre-dose ARA-positivity became negative immediately after dosing only to become positive again months later. Since the ARA assay only detects antibodies unbound to rituximab, as long a free drug is detected, a negative ARA assay would thus be expected even if antibodies are present. Monitoring non-naïve patients at baseline (pre-rituximab) plus months-9 and -12 identified 88% of all positives. ARA-positive patients had non-significantly less immunologic remission, significantly more B-cell reconstitution at 6-months, and significantly less clinical remission at both 6- and 12-months than ARA-negative patients, in contrast to other work cited above (139, 169). They also had more frequent relapses, which occurred earlier. ARA titer had no impact on clinical remission. These results support the authors’ contention that ARA should be monitored at 6 and 9 months in all patients as well as at baseline in those previously exposed.

### B-cell depletion beyond rituximab

Rituximab, a chimeric IgG1 molecule, redistributes CD20 into lipid rafts enhancing complement-dependent cytotoxicity (CDC) (178). Rituximab also kills B-cells by promoting apoptosis (179) and by ADCC (180). Ofatumumab is a fully human IgG1 type 1 anti-CD20 antibody that is more effective in fixing complement than rituximab; however, it was not clinically more effective in treating B-cell malignancies (181). It binds to a different epitope than rituximab, one closer to the cell membrane, facilitating better complement activation (182). Type 2 anti-CD20 antibodies including obinutuzumab, a humanized IgG1 antibody, do not redistribute CD20 into lipid rafts and hence do not effectively induce CDC (183). Obinutuzumab’s Fc region is glycoengineered to lack fucose, which improves binding affinity to FcγRIIIa receptors expressed on effector cells (NK cells, macrophages). Internalization and degradation of antibody-CD20 complexes that occur with rituximab are reduced. ADCC is markedly enhanced (184). Obinutuzumab also promotes non-apoptotic (lysosomal mediated) programmed cell death (185). Although both rituximab and obinutuzumab recognize overlapping epitopes (186), they bind in completely different orientations with obinutuzumab forming terminal complexes inhibiting recruitment of additional molecules and hence reduced complement activation (183).

Humanized monoclonal antibodies, such as obinutuzumab (the only murine component being the hypervariable region) and fully human antibodies (ofatumumab), are in general less immunogenic than chimeric antibodies (rituximab), resulting in a lower incidence of anti-drug antibodies (187), although even fully human antibodies can be immunogenic, directed against the antigen-binding hypervariable region (188). Both ofatumumab and obinutuzumab generally do not cross react with ARA (169, 189). Boyer-Suavet et al. found that the sera from 8 of 10 ARA-positive patients did not inhibit the *in vitro* B-cell cytotoxic potential of ofatumumab and obinutuzumab (169). Three patients with ARA responded to ofatumumab. In a non-randomized, retrospective analysis of 34 ARA-positive patients, Teisseyre et al. compared 19 patients retreated with rituximab to 15 receiving obinutuzumab (12) or ofatumumab (3) (190). At month 6, the combined obinutuzumab/ofatumumab group had significantly greater B-cell depletion (93% versus 35%), immunologic remission (92% versus 56%), and clinical remission (87% versus 37%); at month 12, only clinical remission remained significant (87% versus 42%). Although limited by small numbers, the available data indicate obinutuzumab or ofatumumab should be used in ARA-positive patients requiring further immunosuppression.

Obinutuzumab is being increasingly studied for treating MN regardless of ARA. In a non-randomized but propensity-matched retrospective analysis, Hu et al. compared 21 Chinese patients receiving obinutuzumab (1-gram x2, 15 days apart) to 42 receiving rituximab (rheumatoid arthritis protocol with 34 receiving re-administration at 6 months) and found significantly greater remission at 12-months (20/21 versus 28/42, p=0.03) with obinutuzumab (191). In PLA2R-positive patients (68%), they found significantly lower B-cell counts at 6 months and a trend to greater immunologic remission at 6- and 12-months. By contrast, Cao et al. found no difference in remission rate comparing obinutuzumab (18 patients) and rituximab (21 patients) at 12 months (94.4% versus 85.7%, respectively) in PLA2R-negative Chinese patients (192). Similarly, but in PLA2R-positive Chinese patients, Li et al. found no difference in 6-month clinical or immunologic remission rates between 25 patients given obinutuzumab and 22 patients given rituximab (76% versus 59%, p=0.211, for clinical, and 80% versus 64%, p=0.215, for immunologic, respectively) (193).

Other studies of obinutuzumab evaluated patients with rituximab resistance. Klomjit et al. reported 3 cases of rituximab-resistant PLA2R-positive patients treated with obinutuzumab resulting in 2 partial remissions (194). Sethi et al. gave obinutuzumab and obtained complete remission in 4/10 (40%) and partial remission in 5 (50%), including 4 complete and 2 partial remissions in the 7 previously rituximab-treated patients (6 refractory) (195). Cheng et al. gave obinutuzumab to 30 nephrotic Chinese PMN patients previously treated with rituximab and obtained 86% clinical remissions (196). Li et al. treated 17 rituximab-resistant PLA2R-positive Chinese PMN patients with (197)one or more courses of obinutuzumab and obtained 9 complete remissions and 6 partial remissions by one year, and the other 2 had proteinuria and antibody reductions.

Emerging methodology to selectively deplete autoreactive B-cells involves generation of chimeric autoantibody receptor (CAAR) effector cells, either NK cells (CAAR-NK) (198) or T-cells (CAAR-T) (198, 199). CAAR-T or NK effector cells express part of the specific antigen (e.g., PLA2R) and selectively react with and kill only those B-cells expressing the corresponding antibody as their B-cell receptor. Available data demonstrate effective killing of PLA2R-specific B-cells *in vitro* but not yet *in vivo* either in an experimental model of MN or in humans with the disease. Utilization of CAAR-T cells is costly and labor intensive and requires lymphodepleting chemotherapy prior to infusion (200). Alternatively, CAAR-NK cells can come from umbilical cord blood of healthy individuals and be utilized “off the shelf” for MN patients as necessary (200).

### Anti-B-lineage therapy beyond B-cell depletion

Both B-cell Activating Factor (BAFF) and A Proliferation Inducing Ligand (APRIL) are essential for normal B-cell maturation, class-switching, proliferation, and survival. In one study of 51 PMN patients, both BAFF and APRIL levels were higher in PLA2R-positive patients than in PLA2R-negative patients, with levels like those in proliferative lupus patients (201). In the group of patients with PLA2R-antibody clearance after 6 months of immunosuppression, baseline levels of both BAFF and APRIL were significantly lower than in those without antibody clearance. Similarly, baseline levels were lower in those attaining clinical remission compared to those that did not. Several case reports and a small case series found telitacicept (a dual BAFF/APRIL inhibitor) induced remissions, given alone or with steroids, in 5 of 6 PMN patients (reviewed in (202)). As part of a phase 1/2 dose escalation study, 10 patients with PLA2R-positive PMN (6 with nephrotic range proteinuria) were given povetacicept, also a dual BAFF/APRIL inhibitor, for 48 weeks (203). PLA2R titers decreased by 73% at 12-weeks and by 83% at 48-weeks. Proteinuria decreased by 82% at 48-weeks.

Anti-plasma cell therapy has been tried in PMN. Proteasome inhibition with bortezomib was successful in several case reports of refractory PMN (204–206). The ant-CD38 monoclonal antibody daratumumab was transiently effective in a case report (207). A phase 1b/2a study of felzartamab, another human ant-CD38 monoclonal antibody, involving 31 patients (18 treatment naïve or relapsed, 13 refractory) found reductions of PLA2R-antibody ≥ 50% in 77% of evaluable patients (30.8% complete immunological response at end of study), although partial proteinuria remission occurred in 34.6% (208). No excessive safety concerns emerged.

### Alternative immunosuppression

Calcineurin inhibition (CNI) with cyclosporin or tacrolimus is effective in MN, but is relegated to second or third line because of potential nephrotoxicity (avoid with GFR < 60) and a high relapse rate after discontinuation (209). Also, cyclosporin was inferior (for remission maintenance at 2 years although not at 1 year) when directly compared to rituximab in MENTOR and tacrolimus plus low-dose rituximab was inferior to steroids/cyclophosphamide in STARMEN. Theoretically, CNI can be combined with rituximab at initiation to acutely lower proteinuria and urinary rituximab loss via hemodynamic and direct podocyte effects and thus improve rituximab bioavailability, although no data yet support this.

Mycophenolate mofetil (MMF) has been used in several RCTs. Compared to conservative therapy alone, the addition of MMF monotherapy (2 g/day) for one year did not increase remission or reduce proteinuria (210). MMF (0.5–1 g bid) plus low dose steroids were not inferior to cyclosporin plus low dose steroids in a 48-week trial (211). A combination of prednisone, cyclosporin, and MMF had no difference in remission rates, but there were significantly less adverse events compared to steroids/cyclophosphamide (212). In another study, adding trough-adjusted MMF to tacrolimus did not significantly increase remission rate at 1 year compared to tacrolimus monotherapy, nor did it significantly reduce relapse rate on tacrolimus discontinuation (213). We do not recommend using MMF for PMN unless other agents are not available or not tolerated.

ACTH gel given subcutaneously twice weekly for 1 year was compared to a cyclical 6-month steroid/alkylating agent regimen in a 2-year pilot study which showed no difference in remission rate or adverse events. By contrast, a small prospective cohort study compared synthetic ACTH up to 1 mg twice weekly for 9 months with 46-month follow-up to a historical control group receiving oral cyclophosphamide and prednisone and found significantly less remission and more relapses with synthetic ACTH (214). Many adverse events were noted. We do not recommend ACTH (gel or synthetic) for PMN.

## Discussion

The approach to MN with nephrosis begins with a complete history and physical to evaluate for secondary causes, such as infections, medications/toxins, malignancy, and systemic illnesses. A complete serologic work-up follows, including PLA2R antibody titers by ELISA. In a non-diabetic patient with nephrotic syndrome, eGFR > 60, and an elevated PLA2R antibody titer (both ELISA and IIF), a biopsy can be avoided if no secondary cause is found after thorough evaluation (**Table 1**). The presumptive diagnosis would then be PLA2R-positive PMN, and the patient can be treated as such. If the patient is diabetic, a biopsy should be performed, given the possibility of false positive ELISA in diabetes.

The classification of MN is moving away from separation into primary versus secondary MN towards one based on the defined antigenic target. However, specific associations of such antigens with one or more secondary causes have been elucidated. Furthermore, one antigen may have multiple secondary associations, and a secondary condition may have multiple associated antigens. Most of the defined antigens may also occur as PMN. Hence, we believe separation into PMN versus secondary is indeed important and should be retained. All patients should have an exhaustive search for secondary causes regardless of the antigen, as treatment of the secondary cause would be the first line of therapy. In the absence of a secondary cause, immunosuppression bears consideration, regardless of the targeted antigen (see **Table 5**).

**Table 5:** Key takeaways.

• MN remains the most common cause of nephrotic syndrome in non-diabetic adults • Secondary causes must be excluded • PMN can be divided into PLA2R- positive (70 – 80%) and negative cases • In PLA2R negative cases, numerous other antigens have been defined, most with documented secondary associations; most can also present as PMN • A given antigen can have multiple secondary causes and vice-versa • PLA2R titers are prognostically useful and serve both as a target and a guide to IS effectiveness • IS can be delayed for 3–6 months using NIAT except in very high-risk cases • Rituximab is the first line agent for IS, except for very high-risk cases • Rituximab resistance (20 – 40%) may result from reduced bioavailability, ARA, or chronic scarring • Obinutuzumab or ofatumumab can be used if ARA are detected • Alternative therapies under investigation: BAFF/APRIL inhibition, CAAR-T-cells, anti-PC therapy • Alternating cyclical steroids/cyclophosphamide are indicated for very high-risk cases • Transplantation if ESKD develops but recurrence is common • Recurrence post-transplantation is likely progressive and requires treatment

APRIL: a proliferation inducing ligand; ARA: anti-rituximab antibodies; BAFF: B-cell activating factor; CAART: chimeric autoantibody receptor; MN: membranous nephropathy; IS: immunosuppression; NIAT: non-immunosuppressive anti-proteinuric therapy; PC: plasma cell; PMN: primary membranous nephropathy.

Conservative anti-proteinuric therapy should be utilized in all patients. The major question is the use of immunosuppression given the potential variable course. The important questions are *who* to immunosuppress and *how* to. Patients with sub-nephrotic proteinuria (about 20%) have an excellent prognosis and should not be immunosuppressed initially. However, they require follow-up, since transition to nephrotic syndrome may occur. Otherwise, if nephrotic, the KDIGO-based risk stratification in the best available metric (**Figure 1**) (46).

**Figure 2:**
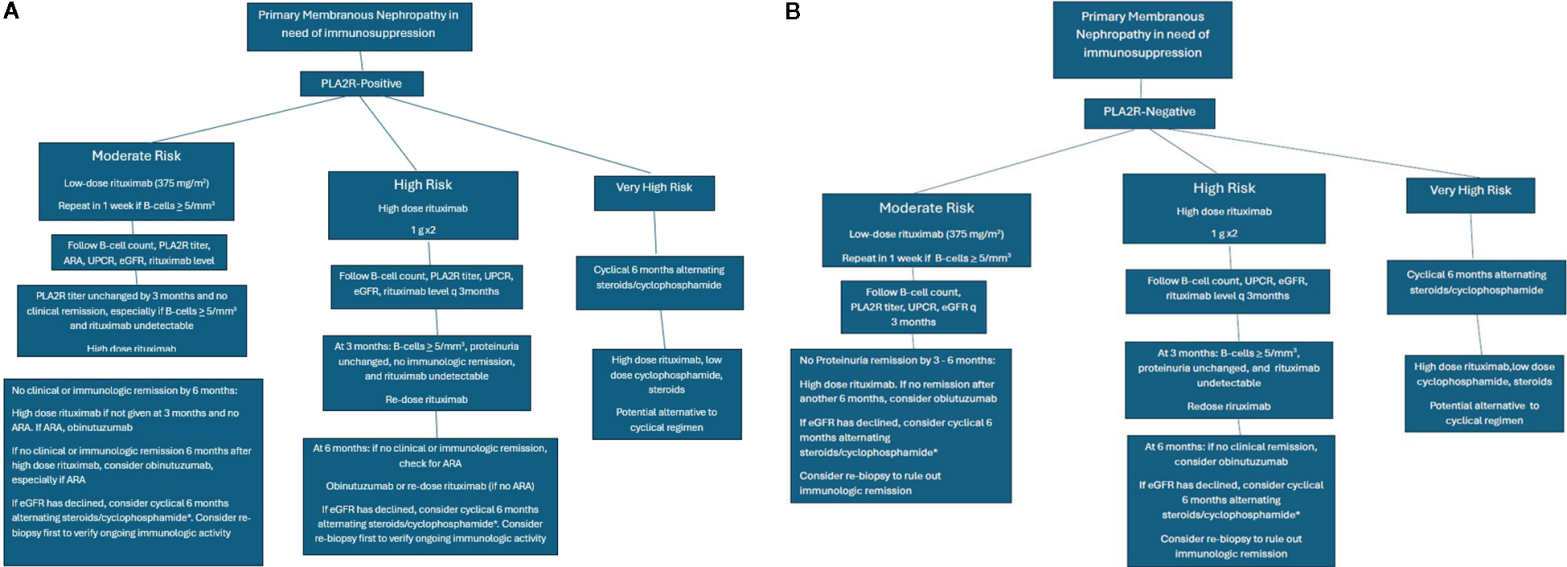
Algorithm for initial approach to immunosuppression in primary membranous nephropathy based on PLA2R-positivity as determined by positive serology. Cases with negative serology but positive tissue stains are otherwise considered PLA2R-positive, but they are considered negative for the purposes of this algorithm since negative PLA2R titers cannot be followed for decline or persistence. **(a)** PLA2R serologically positive. **(b)** PLA2R-negative or PLA2R-positive only by tissue stain. Low-risk cases are not included because they would not be immunosuppressed initially. However, if no remission occurred by 6 months, they would transition to moderate risk and be treated accordingly. In patients previously exposed to rituximab, ARAs should be screened for at baseline and q 3 months. However, the assay can be negative at 3 months but the antibodies may still be present since the assay only measures unbound antibodies. If rituximab naïve, screening can start at 6 months since antibodies would not likely develop by 3 months if previously unexposed. ARA: anti-rituximab antibodies. *Rule out other causes for decline of GFR besides progressive membranous nephropathy. Consider re-biopsy first to verify ongoing immunologic activity.

Low-risk patients should be treated conservatively. For moderate risk-patients, rituximab should be the first choice in our opinion (see **Figure 2**). Equipoise remains as to the optimal regimen. A low-dose regimen is reasonable for moderate risk, such as one 375 mg/m^2^ dose with further doses given, if B-cell depletion (<5/mm^3^) has not occurred within one week (145). Since many patients may respond, it will be cost-effective and reduce the potential for over immunosuppression. Follow-up should include B-cell counts, PLA2R titers (if positive), proteinuria level, and eGFR every three months. In PLA2R-positive patients, failure to decrease titers at 3 months and especially at 6 months should prompt high-dose therapy, unless proteinuria remission has occurred. Failure to achieve clinical remission or at least significant proteinuria reduction by 6 months should also prompt consideration of a high-dose regimen (the rheumatoid arthritis protocol), unless immunologic remission occurs. Alternatively, more potent B-cell depletion with obinutuzumab may be used. If clinical and immunologic remission have not occurred and GFR has decreased, a cyclical steroid/cyclophosphamide should be considered.

In those at high risk, high-dose rituximab remains our first choice. Every 3 months, B-cell levels, rituximab levels, PLA2R-titers (if positive), proteinuria, and eGFR should be measured. B-cell repopulation, and undetectable rituximab levels (and failure to decrease PLA2R-titers if positive) at 3 months should prompt consideration of redosing rituximab (suggests reduced bioavailability from urinary losses) unless proteinuria has remitted. ARA would not be expected this early in rituximab-naïve patients but could occur by 3 months if previously exposed and in that case should be measured.

Although lack of remission at 6-months implies rituximab resistance, remission may occur after 6-months (144), even after one year (215). Therefore, the optimal time to define resistance is uncertain, but 6 months seems reasonable to guide alterations in therapy. Resistance may result from non-immunologically mediated chronic scarring, reduced bioavailability, or ARA.

In PLA2R-positive patients, immunologic remission with persisting proteinuria suggests chronic scarring and non-ongoing immunologic activity. In PLA2R-negative patients, a repeat biopsy would be necessary. Reduced rituximab bioavailability should be suspected, if undetectable rituximab levels and/or B-cell repopulation occurred at 3 months. Options for reduced bioavailability include an additional course of high-dose rituximab versus a switch to obinutuzumab. If ARA are detected with clinical resistance and persisting PLA2R-antibody titers, we favor obinutuzumab given the lack of cross-reactivity and reported clinical effectiveness. Ofatumumab is also acceptable.

If no obvious cause of rituximab resistance is apparent and clinical and immunologic remission has not occurred at 6-months in high-risk cases, a second course of high-dose rituximab should be considered. Obinutuzumab or ofatumumab are alternatives. A steroid/cyclophosphamide-based regimen would be favored if eGFR has decreased. RCTs are needed to address these issues, as well as whether alternative immunosuppression such as CNI should be considered.

Continued follow-up is required if clinical or immunologic remission has occurred, given that about one-third may relapse, especially with only partial remission. Aside from proteinuria, PLA2R-antibodies should be monitored every three months if applicable, since antibody reemergence precedes clinical relapses.

Whether epitope spreading in PLA2R-positive patients can be used to personalize rituximab therapy in patients at moderate to high risk of progression is a possibility based on the RCT of Brglez (159) outlined above; however, it remains to be validated in another population, especially given the conflicting results regarding epitope spreading.

For very high-risk cases, an alternating steroids/cyclophosphamide regimen has the best long-term outcome data and is the KDIGO recommended regimen. This would be our first choice if the patient is agreeable. A possible alternative is the high-dose rituximab/low-dose cyclophosphamide/short-course steroid regimen of Zonozi et al (160). It remains uncertain if a high-dose rituximab protocol alone, followed by repeat dosing at 3 or 6 (at the latest) months based on rituximab levels, B-cell levels, and PLA2R-antibody titers, is also a viable alternative for very high-risk cases. The role of obinutuzumab and ofatumumab are also uncertain. RCTs are clearly needed.

For those unable to receive or refractory to these first line therapies, alternatives include CNI, ACTH gel, and MMF. Of these, CNI has the best support, with the caveats that there is nephrotoxicity and high relapse rate upon discontinuation. CNI use should probably be limited to moderate risk patients unable to receive or tolerate rituximab. We do not recommend ACTH or MMF.

There are insufficient data to make recommendations on emerging therapies, such as APRIL and BAFF inhibition; CAAR T-cell (or NK-cell) selective, antigen-specific B-cell depletion; or anti-plasma cell therapy (proteasome inhibition or anti-CD38 monoclonal antibodies). The role of complement inhibitors remains to be determined.

Transplantation remains the optimal therapy for PMN progressing to ESKD. Recurrence is common and usually occurs within the first year, although it may occur years later. Proteinuria mandates biopsy. In PLA2R-positive patients, persisting or rising titers should prompt a biopsy as should reemerging titers, regardless of proteinuria. Since the original disease progressed to ESKD and recurred despite immunosuppression post-transplantation, SR is unlikely and therapy should be considered without waiting. Rituximab would be our choice.

There are obvious limitations to this review since it is narrative and not systematic, introducing bias potential in included studies. Although RCTs were highlighted, most studies are retrospective and observational and thus are mainly hypothesis generating. There are geographical limitations, with studies often limited to one specific population which hampers generalization. However, we feel our conclusions are reasonable given the data presented.

## Glossary

ACTH: adrenocorticotropin hormone
ADCC: antibody dependent cellular cytotoxicity
ANCA: anti-neutrophil cytoplasmic autoantibodies
APRIL: a proliferation inducing ligand
ARA: anti-rituximab antibodies
BAFF: B-cell activating factor
CAAR-T/CAAR-NK: chimeric autoantibody reactive T-cell or NK-cell, respectively
CDC: complement dependent cytotoxicity
CIDP: chronic inflammatory demyelinating polyneuropathy
CNI: calcineurin jinhibition
CNTN1: contactin 1
CTLD: C-type lectin domain
CysR: cysteine-rich domain
eGFR: estimated glomerular filtration rate
ELISA: enzyme linked immunosorbent assay
EM: electron microscopy
ESKD: end-stage kidney disease
EXT1/2: exostosin 1 and 2
FAT1: protocadherin FAT1
FcγRIIb: Fc gamma receptor IIb
FcRn: neonatal Fc receptor
FNII: fibronectin type-II domain
FSGS: focal segmental glomerulosclerosis
GBM: glomerular basement membrane
GFR: glomerular filtration rate
GVHD: graft versus host disease
HSCT: hematopoietic stem cell transplantation
HTRA1: high temperature requirement A serine peptidase
IFM: immunofluorescence microscopy
IIF: indirect immunofluorescence assay
KDIGO: Kidney Disease Improving Global Outcomes consortium
LM: light microscopy
LM/MS: laser microdissection tandem mass spectrometry
LMN: lupus membranous nephropathy
MCD: minimal change disease
MMF: mycophenolate mofetil
MN: membranous nephropathy
MPO: myeloperoxidase
NCAM1: neural cell adhesion molecule 1
NDNF: neuron derived neurotrophic factor
NELL-1: neural epidermal growth factor like-1
NIAT: non-immunosuppressive anti-proteinuric therapy
NTNG1: netrin G1
NSAID: non-steroidal anti-inflammatory drug
PCDH7: protocadherin 7
PCSK6: Proprotein convertase subtilisin/kexin type 6
PLA2R: phospholipase A2 receptor 1
PMN: primary membranous nephropathy
RAS: renin angiotensin system
RCT: randomized controlled trial
ROC: receiver operator characteristic curve
RU: relative unit
Sema3B: semaphoring 3B
SGLT2: sodium glucose cotransporter 2
SNP: single nucleotide polymorphism
SR: spontaneous remission
TGFBR3: transforming growth factor B receptor 3
THSD7A: thrombospondin type 1 domain-containing 7A
WB: western blot
